# RhoA/ROCK signalling activated by ARHGEF3 promotes muscle weakness via autophagy in dystrophic *mdx* mice

**DOI:** 10.1002/jcsm.13278

**Published:** 2023-06-13

**Authors:** Jae‐Sung You, Yongdeok Kim, Soohyun Lee, Rashid Bashir, Jie Chen

**Affiliations:** ^1^ Department of Cell and Developmental Biology University of Illinois at Urbana‐Champaign Urbana Illinois USA; ^2^ Department of Bioengineering University of Illinois at Urbana–Champaign Urbana Illinois USA; ^3^ Nick J. Holonyak Micro and Nanotechnology Laboratory University of Illinois at Urbana–Champaign Urbana Illinois USA; ^4^ Department of Materials Science and Engineering University of Illinois at Urbana–Champaign Urbana Illinois USA; ^5^ Department of Mechanical Science and Engineering University of Illinois at Urbana–Champaign Urbana Illinois USA; ^6^ Department of Biomedical and Translational Sciences Carle Illinois College of Medicine Urbana Illinois USA

**Keywords:** *Mdx*, Engineered muscle, Force, Regeneration, XPLN, Chloroquine

## Abstract

**Background:**

Duchenne muscular dystrophy (DMD), caused by dystrophin deficiency, leads to progressive and fatal muscle weakness through yet‐to‐be‐fully deciphered molecular perturbations. Emerging evidence implicates RhoA/Rho‐associated protein kinase (ROCK) signalling in DMD pathology, yet its direct role in DMD muscle function, and related mechanisms, are unknown.

**Methods:**

Three‐dimensionally engineered dystrophin‐deficient *mdx* skeletal muscles and *mdx* mice were used to test the role of ROCK in DMD muscle function *in vitro* and *in situ*, respectively. The role of ARHGEF3, one of the RhoA guanine nucleotide exchange factors (GEFs), in RhoA/ROCK signalling and DMD pathology was examined by generating *Arhgef3* knockout *mdx* mice. The role of RhoA/ROCK signalling in mediating the function of ARHGEF3 was determined by evaluating the effects of wild‐type or GEF‐inactive ARHGEF3 overexpression with ROCK inhibitor treatment. To gain more mechanistic insights, autophagy flux and the role of autophagy were assessed in various conditions with chloroquine.

**Results:**

Inhibition of ROCK with Y‐27632 improved muscle force production in 3D‐engineered *mdx* muscles (+25% from three independent experiments, *P* < 0.05) and in mice (+25%, *P* < 0.001). Unlike suggested by previous studies, this improvement was independent of muscle differentiation or quantity and instead related to increased muscle quality. We found that ARHGEF3 was elevated and responsible for RhoA/ROCK activation in *mdx* muscles, and that depleting ARHGEF3 in *mdx* mice restored muscle quality (up to +36%, *P* < 0.01) and morphology without affecting regeneration. Conversely, overexpressing ARHGEF3 further compromised *mdx* muscle quality (−13% vs. empty vector control, *P* < 0.01) in GEF activity‐ and ROCK‐dependent manner. Notably, ARHGEF3/ROCK inhibition exerted the effects by rescuing autophagy which is commonly impaired in dystrophic muscles.

**Conclusions:**

Our findings uncover a new pathological mechanism of muscle weakness in DMD involving the ARHGEF3‐ROCK‐autophagy pathway and the therapeutic potential of targeting ARHGEF3 in DMD.

## Introduction

Duchenne muscular dystrophy (DMD) is a severe and common form of muscular dystrophy, with an estimated incidence of about 1 in 3800 male births, and is caused by diverse inherited and spontaneous mutations in the X‐linked dystrophin gene.^1^ The loss of dystrophin results in progressive muscle weakness and premature death before the age of 30 years without ventilatory support.^2^ DMD muscle fibres exhibit high injury susceptibility that causes necrotic degeneration followed by regeneration that compensates for the loss of myofibres.^3^ Regeneration requires a highly coordinated myogenesis process consisting of muscle stem cell proliferation, differentiation, and myoblast fusion, but in DMD, the efficiency of this process is known to be compromised by dystrophin deficiency‐mediated muscle stem cell dysfunction.^4^, ^5^ Studies have shown great promise in enhancing or correcting the myogenic potential of muscle stem cells for improving muscle regeneration and function in DMD.^4^, ^5^

While regeneration is essential to support the quantity of DMD muscle, it does not entirely compensate for muscle weakness in DMD due to the loss of muscle quality, a term defined as the capacity of a given muscle to generate force (i.e. specific muscle force).^6^ The quality of DMD muscle is fundamentally impacted by the loss of force transmission in myofibres, and this occurs before the onset of major myofibre necrosis (<2 weeks in *mdx* mice, a model of mild DMD pathology) and the introduction of other non‐muscle factors affecting muscle quality, such as fibrosis and fat infiltration.^7^ Hence, in addition to regeneration, developing therapies that improve muscle‐intrinsic force transmission or myofibre quality is key to treating muscle weakness in DMD.

While our understanding of the molecular regulators of the myogenic process in DMD is advancing, information on the role of these regulators in muscle quality and function in DMD is still limited. One example involves the small GTPase RhoA and its major effector, Rho‐associated protein kinase (ROCK). ROCK is a member of the AGC kinase family, which regulates a wide range of cellular activities through actin cytoskeleton reorganization and is implicated in various pathologic conditions, with some of its inhibitors already approved for clinical use.^8^ It has been reported that RhoA/ROCK signalling is activated in *mdx* skeletal muscle and that inhibiting RhoA/ROCK signalling promotes muscle differentiation of myogenic progenitor cells (MPCs) lacking dystrophin and its homologue utrophin (*mdx*/UKO).^9^, ^10^, ^11^ Inhibition of RhoA/ROCK signalling is also known to increase myofibre quantity in *mdx* and *mdx*/UKO muscles.^9^, ^10^, ^11^ Despite the implication of its involvement in muscle regeneration in DMD, however, whether and how RhoA/ROCK signalling regulates muscle quality and function is not well understood, making its clinical significance less clear. It is also unknown how this potentially important pathway is regulated in DMD.

In this study, by using 3D‐engineered *mdx* muscle tissues *in vitro* and *mdx* mice *in vivo*, we uncover a critical role of ROCK in regulating DMD muscle quality and identify ARHGEF3 as the main driver of RhoA/ROCK activation and muscle weakness. Unexpectedly, this function of ARHGEF3 and ROCK is independent of muscle regeneration and instead dependent on autophagy.

## Materials and methods

### Animals

All animal experiments were performed in agreement with protocols approved by the Institutional Animal Care and Use Committee at the University of Illinois at Urbana‐Champaign (#19255). *Dystrophin* and *Arhgef3* double‐knockout (*mdx*/AKO) mice were generated first by crossing *mdx* female mice (RRID:IMSR_JAX:001801) with *Arhgef* ^*−/−*^ male mice,^12^ then by serially crossing offspring with *Arhgef* ^*+/−*^ mice. All mouse lines were maintained on a C57BL/6N background from the same pedigree and genotyped for *dystrophin* and *Arhgef3* alleles by PCR as previously described.^12^, ^13^ Mice of the same age between 5‐ to 9‐week‐old were randomly allocated to the various experimental groups and anaesthetised with isoflurane during all surgical procedures and at the end of the experiments for euthanasia. Except for some phenotyping experiments where we used both males and females (indicated in figure legends), all experiments were performed with male mice. Animals were housed in a room maintained at 23°C with a 12‐h light/dark cycle and received a pellet diet and water ad libitum.

### Skeletal muscle transfection

Transfection of TA muscle was performed by electroporation as previously described^14^ with slight modifications. Briefly, an incision was made on the skin along the TA muscle, and a 30 μL of plasmid DNA solution containing 60 μg of either pCMV‐Myc‐ARHGEF3, pCMV‐Myc‐ARHGEF3‐L269E,^15^ or pcDNA3 empty vector was injected into the distal end of the TA muscle with a 27‐gauge needle. Immediately after the injection, eight 20‐ms square‐wave electric pulses were delivered to the muscle at 1 Hz with a field strength of 50 V/cm through two stainless steel pin electrodes (1‐cm gap) connected to an ECM 830 electroporation unit (BTX/Harvard Apparatus). The incised skin was then closed with a 3‐0 polysorb suture.

### *In situ* muscle force measurement

Muscle force analyses and eccentric contractions were performed *in situ* using a 1300A Whole‐Animal System (Aurora Scientific) as described previously.^12^ Briefly, the distal tendon of the TA muscle was connected to the lever arm of the force transducer through a 3–0 suture line. The muscle was electrically stimulated with 0.2‐ms square‐wave pulses at 0.2 mA and adjusted to optimal muscle length where maximal twitch force was produced. The maximum isometric tetanic force was determined in the frequency range of 50–200 Hz with 300‐ms pulse duration, with each contraction separated by a 1‐minute rest. For eccentric contractions, the TA muscle was lengthened to 1.2 fibre length (0.6 × optimal muscle length) at a velocity of 1.5 fibre length/s and held for 200 ms before being returned to its optimal length at the same velocity. The muscle was stimulated 100 ms prior to and during the lengthening period at 100 Hz. Specific isometric twitch/tetanic force was calculated by dividing maximal isometric twitch/tetanic force by physiological cross‐sectional area [muscle mass/(fibre length × muscle density 1.06 g/cm^3^)].

### Isolation of MPCs and cell culture

MPCs were isolated from 2‐week‐old wild‐type (WT) and *mdx* female mice using a modified version of the previously described methods.^16^, ^17^ Briefly, hindlimb muscles were minced and enzymatically digested using collagenase (type 2, Worthington Biochemical Inc.) and dispase (type 2, Roche Diagnostics).^16^ The digestion solution containing muscle tissue fragments was seeded on tissue culture plates coated with 0.7–0.8 mg/mL Matrigel (Corning) to allow the outgrowth of MPCs from muscle explants.^17^ Expanded single cells were collected through a 40 μm cell strainer and preplated twice for 1 h each on plates coated with 0.01% collagen (type I from calf skin, Sigma‐Aldrich) to remove adherent non‐MPCs. The purity of MPCs reached near 100% by serial passaging with 15 min incubation of PBS that preferentially detached MPCs from collagen‐coated plates. The medium used for MPC proliferation (PM) was composed of high glucose DMEM, 30% fetal bovine serum, 1% penicillin–streptomycin, and 5 ng/mL bFGF. To induce differentiation, MPCs were seeded on Matrigel‐coated plates, grown to 100% confluence, and cultured in differentiation medium (DM) composed of high glucose DMEM, 2% horse serum, and 1% penicillin–streptomycin.

### Construction of *in vitro* 3D skeletal muscle

Fabrication of 3D skeletal muscle and hydrogel structure was described previously in detail. Briefly, the cell‐matrix solution consisted of 1 × 10^7^ cells/mL of MPCs, 30% (vol/vol) Matrigel, 4 mg/mL fibrinogen (Sigma‐Aldrich), and 0.5 U of thrombin (Sigma‐Aldrich)/mg of fibrinogen was seeded in a polydimethylsiloxane ring mould with 5 mm/6.6 mm for inner/outer diameters. After 2 h, the PM supplemented with 1 mg/mL aminocaproic acid (Sigma‐Aldrich) was added to the ring mould and changed every other day. After 7 days, compacted muscle rings were transferred into the 3D‐printed hydrogel cantilevers and cultured with the DM supplemented with 1 mg/mL of aminocaproic acid and 0.5 ng/mL of insulin‐like growth factor‐1 (Sigma‐Aldrich). The 3D hydrogel cantilever structure was designed in Solidworks (Figure S1) and fabricated using the digital light processing 3D printer (PICO2, Asiga) and a printing resin solution containing 20% (vol/vol) polyethylene glycol diacrylate M.W. 700 (Sigma‐Aldrich), 1 mg/mL of lithium phenyl‐2,4,6‐trimethylbenzoylphosphinate (Sigma‐Aldrich), and 0.4 mg/mL of Sunset Yellow FCF (Sigma‐Aldrich). Myofibre alignment of the 3D muscle ring was confirmed by scanning electron microscopy (FEI Quanta FEG 450).

### *In vitro* force measurement

*In vitro* muscle ring contractions were elicited by 50‐ms bipolar electric pulses at 1 Hz with a field strength of 10 V/cm using a custom‐built electrical setup^19^ and recorded by a portable digital microscope camera (Dino‐Lite) on the top of the muscle ring. The movement of the cantilever pillar was tracked using the software Tracker (https://physlets.org/tracker). Muscle force was calculated using the Euler‐Bernoulli beam bending theory as follows.

$$F=\;3\mathrm{EId}/\left[a^{3}\left(1+3b/2a\right)\right]$$

Where *I* is the moment of inertia, *d* is the deflection on the cantilever, *a* is the length between the bottom surface and the muscle, and *b* is the distance between the muscle and the top of the cantilever. *E* represents the Young's modulus of the cantilever, which was measured to be 270 kPa.

### Drug treatments

For *in vivo* experiments, Y‐27632 dihydrochloride (in DMSO) and chloroquine diphosphate (Sigma‐Aldrich) (in water) stock solutions were diluted in PBS and injected intraperitoneally into mice at the final concentrations of 5 and 50 mg/kg body weight, respectively. These injections were given every 24 h and/or 1 h prior to analyses or sample collection. Control mice were injected with an equivalent amount of vehicle diluted in PBS. For *in vitro* experiments, 10 μM of Y‐27632 dihydrochloride or an equal amount of DMSO was incubated in culture media for the indicated periods. The drug‐containing media was changed every 24 h.

### Biochemical analyses

Additional materials and methods regarding biochemical analyses, including Antibodies, Immunohistochemistry, Immunofluorescence, Western Blotting, RhoA activity assay, and Quantitative PCR, are provided in the supporting informatioin.

### Statistical analysis

All values were presented as mean ± SEM unless otherwise noted, with individual data points shown in graphs (the number of the points represents n). The sample size for each experiment was determined based on previous publications and preliminary data. A quantified sample value that deviated more than three times SD from the mean in a given group was removed as an outlier. Statistical significance (*p* < 0.05) was determined by two‐tailed paired (when comparing to contralateral controls) or unpaired *t*‐tests for single comparisons or one‐ or two‐way ANOVA followed by the Student–Newman–Keuls post hoc test for multiple comparisons. All statistical analyses, including assumption tests, were performed using SigmaPlot 14.0 (https://systatsoftware.com/).

## Results

### ROCK inhibition improves *mdx* muscle function *in vitro* independently of differentiation

**Figure 1:**
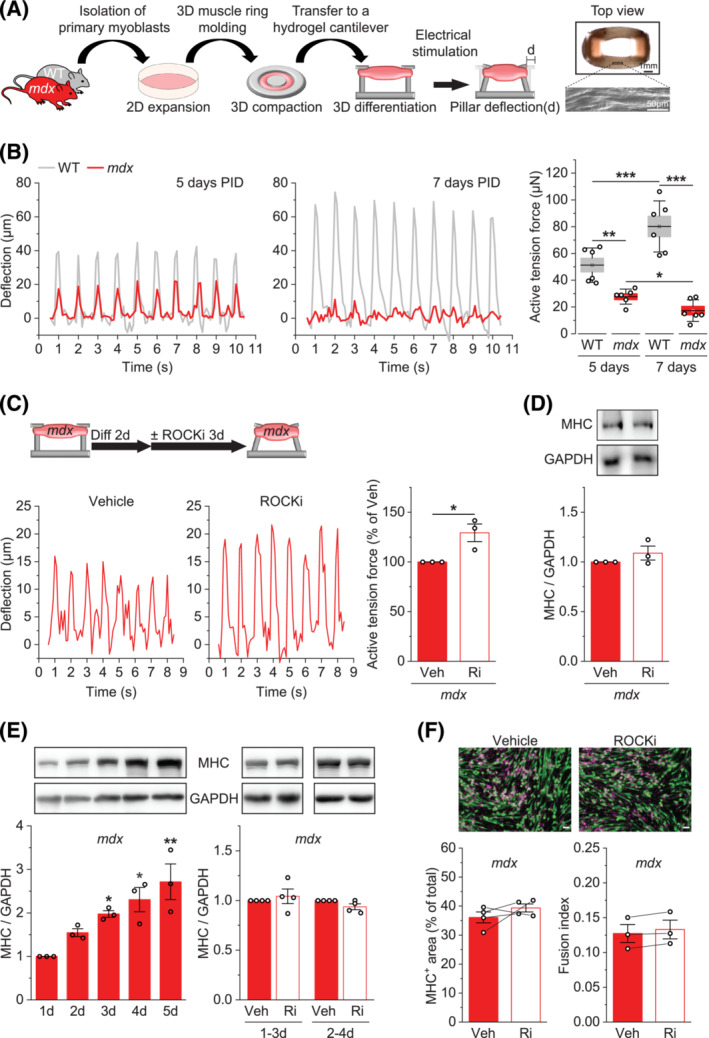
ROCK inhibition improves *mdx* muscle function *in vitro* independently of differentiation. (A) A schematic of constructing 3D‐engineered skeletal muscle using myogenic progenitor cells (MPCs) from WT and *mdx* mice, with a top view of the muscle ring and its magnification by scanning electron microscopy showing aligned muscle fibres. (B) Engineered 3D WT and *mdx* skeletal muscles were electrically stimulated at 1 Hz on day 5 and day 7 post‐induction of differentiation (PID), and the resulting deflection of the cantilever pillar was recorded and converted to active tension force. (C) Differentiating 3D *mdx* skeletal muscles were treated with Y‐27632 (Ri) or a control vehicle (Veh) from day 2 PID and electrically stimulated on day 5 PID to acquire deflection and active tension force data as in (B). (D) Muscle samples from (C) were analysed for protein expression of myosin heavy chain (MHC) and GAPDH. *(E) mdx* MPCs were differentiated on a 2D plate for up to 5 days (left), with some cells treated with Y‐27632 (Ri) or a control vehicle (Veh) during the last 2 days of differentiation (right). The cells were analysed for protein expression of myosin heavy chain (MHC) and GAPDH. (F) Differentiating 2D *mdx* MPCs were treated with Y‐27632 (Ri) or a control vehicle (Veh) from day 2 to day 5 PID as in (C) and analysed for MHC^+^ area and fusion index. Data are presented as mean ± SEM (box) and SD (whisker) (B) or ± SEM (C–F) with individual data points from each muscle (B) or independent experiment (C–F). *P < 0.05, **P < 0.01, ***P < 0.001 by two‐way ANOVA (B) or two‐tailed paired *t*‐test (C–F).

Despite the implication of RhoA/ROCK signalling in myogenic differentiation of dystrophin‐deficient MPCs, whether this is ultimately linked to the regulation of contractile muscle function is unknown. To directly address this issue, we engineered 3D in‐vitro skeletal muscle tissue using mouse MPCs and a 3D‐printed cantilever structure (Figures 1A and S1). As shown in Figure 1B and Movie S1, electrical stimulations successfully induced contractions of the 3D skeletal muscle ring construct, which was tracked by the deflection of the 3D‐printed cantilever pillars that hold the muscle ring. Active tension force calculated from the deflection increased from day 5 to day 7 post‐induction of differentiation (PID) in WT muscle constructs. However, in *mdx* constructs, the force was nearly half compared with WT on day 5 PID and became even lower over the next 2 days (Figure 1B and Movie S1), mimicking muscle intrinsic and progressive weakness in DMD. When we treated *mdx* muscle constructs with the ROCK inhibitor Y‐27632 during differentiation, the active tension force was significantly improved (Figure 1C and Movie S2). Much to our surprise, this improvement in force occurred in the absence of any increase in the expression of MHC protein, a late marker of muscle differentiation (Figure 1D). These results suggest that ROCK negatively regulates *mdx* muscle contractility by directly impacting force transmission and not differentiation. To further confirm this, we performed a standard 2D muscle differentiation assay in which *mdx* MPCs progressively differentiated for at least 5 days (Figure 1E, left panel). Consistent with our 3D muscle data, treating these cells with the ROCK inhibitor did not affect muscle differentiation measured by MHC expression (Figure 1E, right panel), MHC^+^ area, and fusion index (Figure 1F).

### ROCK inhibition improves *mdx* muscle quality and function *in vivo*

**Figure 2:**
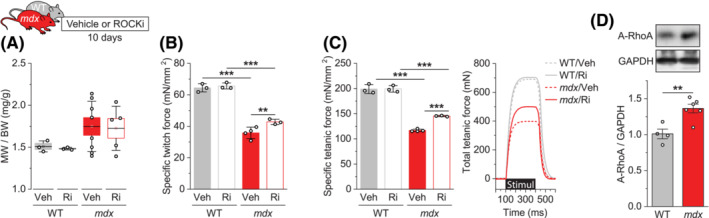
ROCK inhibition improves *mdx* muscle quality and function *in vivo*. (A–C) Tibialis anterior (TA) muscles from 5‐week‐old WT and *mdx* male mice treated with Y‐27632 (Ri) or vehicle (Veh) for 10 days were analysed for (A) muscle weight (MW) to body weight (BW) ratio, (B) specific isometric twitch force, and (C) specific isometric tetanic force. (D) TA muscles from 5‐week‐old WT and *mdx* male mice were analysed for the amount of GTP‐bound active RhoA and GAPDH. Data are presented as mean ± SEM (box) and SD (whisker) (A) or ± SEM (B−D) with individual data points from each mouse. **P < 0.01, ***P < 0.001 by two‐way ANOVA (B, C) or two‐tailed unpaired *t*‐test (D).

Based on our *in vitro* data, ROCK appears to have a negative role in *mdx* muscle function via differentiation‐independent mechanisms that involve control of force transmission or muscle quality. We next probed the role of ROCK *in vivo* by treating *mdx* mice with Y‐27632 for 10 days and measuring the mass and function of the tibialis anterior (TA) muscle at 5 weeks of age. It is noteworthy that the lower hindlimb muscles (e.g. TA) of *mdx* mice at this young age undergo an active regeneration process but do not yet suffer from fibrosis or fat infiltration.^9^, ^20^, ^21^ Thus, muscle mass and specific muscle force at this stage can indicate the level of muscle regeneration and myofibre quality, respectively. The results showed that muscle mass in *mdx* mice tended to increase compared with WT mice but was not influenced by the treatment of the ROCK inhibitor, suggesting that ROCK may not play a major role in muscle regeneration in *mdx* mice (Figure 2A). However, we found that specific muscle force, both twitch and tetanic, was drastically reduced in *mdx* muscles, and ROCK inhibition partially, but significantly, alleviated this loss of muscle quality and function (Figures 2B,C). These results are consistent with our data from 3D‐engineered muscles and implicate ROCK in the pathogenesis of myofibre dysfunction in *mdx* mice. In support of this point, we also observed an increased amount of active RhoA (activator of ROCK) in *mdx* muscles (Figure 2D).

### ARHGEF3 mediates activation of RhoA/ROCK signalling in *mdx* muscles

**Figure 3:**
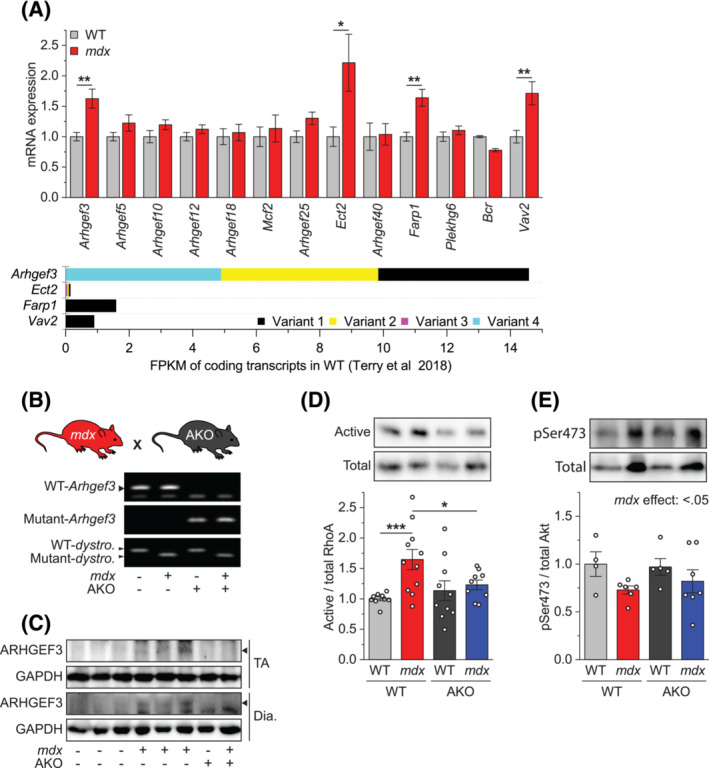
ARHGEF3 mediates activation of RhoA/ROCK signalling in *mdx* muscles. (A) Tibialis anterior (TA) muscles from 5‐week‐old WT and *mdx* male mice were analysed for mRNA expression of 13 RhoA GEFs (upper) (*n* = 5–7 mice per group). The FPKM of coding transcripts of *Arhgef3*, *Ect2*, *Farp1*, and *Vav2* from MuscleDB^23^ (lower). (B) *Dystrophin* and *Arhgef3* double‐knockout (*mdx*/AKO) mice generated from *mdx* and *Arhgef3* KO (AKO) mice were genotyped using tail DNA. (C–E) TA muscles from 5‐week‐old WT, *mdx*, AKO, and *mdx*/AKO male mice were analysed for protein expression of ARHGEF3 and GAPDH (C), RhoA GEF activity (D), and phosphorylation of Akt (E). Data are presented as mean ± SEM (with individual data points from each mouse in (D) and (E)). *P < 0.05, **P < 0.01, ***P < 0.001 by two‐tailed unpaired *t*‐test (A) or two‐way ANOVA (D, E).

In humans, there are at least 28 RhoA guanine nucleotide exchange factors (GEFs) that can activate RhoA and subsequently ROCK signalling,^22^ and all of these were found to be transcriptionally expressed in mouse skeletal muscle (Expression Atlas; https://www.ebi.ac.uk/gxa/). Of note, some of these RhoA GEFs can also activate RhoB and/or RhoC, but there is no RhoB‐ or RhoC‐specific RhoGEF. We wondered which RhoA GEF might be responsible for the regulation of RhoA/ROCK signalling in *mdx* muscles. Based on structural homology, those 28 RhoA GEFs can be divided into 13 classes,^22^ and we examined gene expression levels of 13 RhoA GEFs, each representing one of the different classes. As shown in Figure 3A, four RhoA GEFs were significantly increased in *mdx* muscles, which include *Arhgef3*, *Ect2*, *Farp1*, and *Vav2*. We compared the abundance of all coding splice variants of these genes from the rodent muscle RNAseq database, MuscleDB,^23^ and found that *Arhgef3* is at least 9 times more abundant than the others in WT muscles (Figure 3A). We also obtained similar results when using Ct values from our qPCR analysis (Figure S2). These results suggest that, in the *mdx* muscle, ARHGEF3 is the most abundant among the GEFs and may have contributed to the regulation of RhoA/ROCK signalling and downstream phenotypes. To directly test this idea, we generated *dystrophin* and *Arhgef3* double‐knockout (*mdx*/AKO) mice (Figure 3B). This mouse line showed no detectable phenotypic abnormality, including body weight (Figure S3). We first analysed protein expression of ARHGEF3 in *mdx* and *mdx*/AKO skeletal muscles using our custom‐made antibody^15^ which, to the best of our knowledge, is the only one validated for specificity, although its avidity is low. As shown in Figure 3C, ARHGEF3 protein was elevated in *mdx* muscles, and it was not detected in *mdx*/AKO muscles. We also found that KO of ARHGEF3 prevented an increase in RhoA GEF activity (active RhoA per total RhoA) in *mdx* muscles (Figure 3D), which indicates that ARHGEF3 is indeed a major RhoA GEF that activates RhoA/ROCK signalling in *mdx* muscles. Independent of its GEF function, ARHGEF3 is known to play a role in inhibiting Akt signalling which is also implicated in DMD pathology^15^, ^24^; however, depletion of ARHGEF3 did not affect Akt phosphorylation in *mdx* muscles (Figure 3E).

### ARHGEF3 depletion improves *mdx* muscle quality and function independently of regeneration

**Figure 4:**
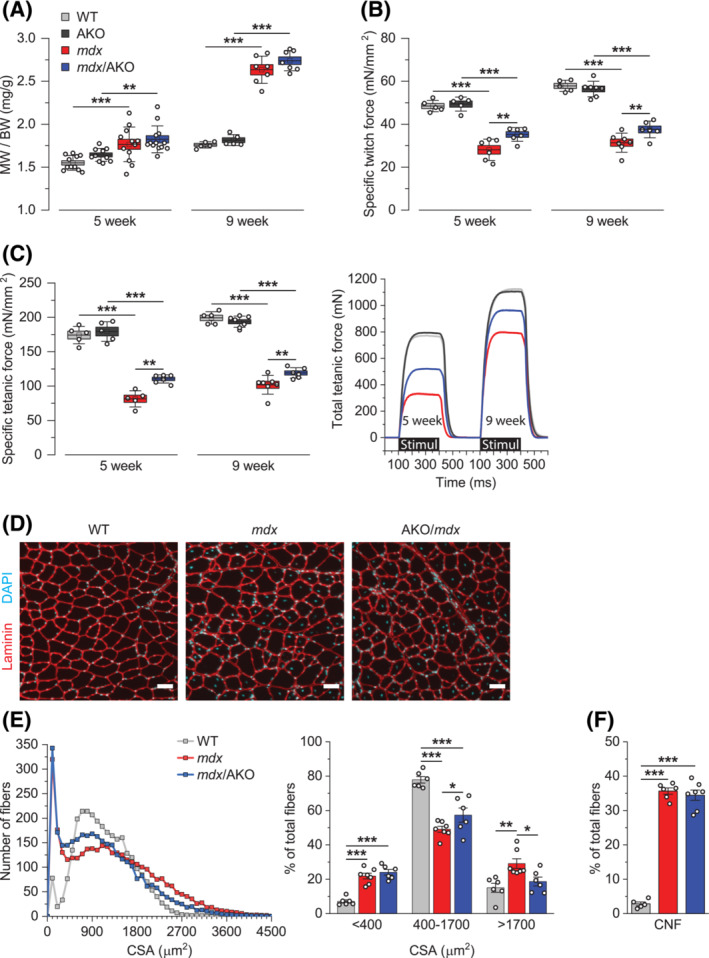
ARHGEF3 depletion improves *mdx* muscle quality and function independently of regeneration. (A–C) Tibialis anterior (TA) muscles from WT, *mdx*, *Arhgef3* KO (AKO), and *mdx*/AKO male mice were analysed for (A) muscle weight (MW) to body weight (BW) ratio, (B) specific isometric twitch force, and (C) specific isometric tetanic force. (D) TA muscle cross‐sections from 5‐week‐old WT, *mdx*, and *mdx*/AKO male mice were stained for laminin and DAPI. (E, F) Images from (D) were analysed for (E) the cross‐sectional area (CSA) of entire myofibres and (F) the proportion of centrally nucleated myofibres. Data are presented as mean ± SEM (box) and SD (whisker) (A–C) or ± SEM (E, F) with individual data points from each mouse. *P < 0.05, **P < 0.01, ***P < 0.001 by two‐way ANOVA (A–C) or one‐way ANOVA (E, F).

Next, we asked if ARHGEF3 is also involved in DMD phenotypes in *mdx* mice. As expected, dystrophin deficiency in *mdx* mice led to an increase in muscle mass compared with WT mice at 5 weeks, and this increase became more pronounced at 9 weeks of age, most likely due to robust regeneration; similar to ROCK inhibition, ARHGEF3 depletion did not affect the increase in muscle mass (Figure 4A). Importantly, specific muscle force and function, which were severely impaired in *mdx* mice, were significantly rescued by ARHGEF3 depletion throughout the young age (Figures 4B,C), and these results were not sex‐specific (Figure S4). The effect of ARHGEF3 depletion in *mdx* mice is also unlikely to be due to different levels of injury or inflammation. For example, ARHGEF3 KO did not prevent *mdx* muscles from eccentric contraction‐induced force drop (Figure S5), a common method to assess muscle injury susceptibility.^3^ ARHGEF3 depletion also did not affect mRNA levels of key inflammatory markers in *mdx* muscles (Figure S6), which is known to increase in DMD and impact muscle function.^25^

To further determine the role of ARHGEF3 in *mdx* pathology, we analysed the size of and the presence of central nuclei, a key feature of regenerating myofibres, in all myofibres in the TA muscle cross‐section using Open‐CSAM software (Figures 4D and S7). Previously we showed that muscles with ARHGEF3 KO alone were indistinguishable from WT muscles regarding myofibre size and central nuclei.^12^ As shown in the histogram in Figure 4E, *mdx* myofibres were highly heterogeneous in size, with a higher number of small (<400 μm^2^) and large (>1700 μm^2^) myofibres and a lower number of regular‐sized (400–1700 μm^2^) myofibres when compared with WT. This pattern of distribution reflects the formation of new myofibres (i.e. regeneration) and pathological hypertrophy of existing or repairing myofibres.^26^, ^27^ Notably, the depletion of ARHGEF3 partially normalized the population of regular‐sized myofibres by preventing the increase in the number of enlarged myofibres (Figure 4E, right panel). On the other hand, ARHGEF3 depletion did not change the population of small myofibres (Figure 4E, right panel) or the number of myofibres with central nuclei (Figure 4F). Combined, our results suggest that ARHGEF3 plays a critical role in the dysregulation of *mdx* myofibres independently of regeneration.

### ARHGEF3 regulates *mdx* muscle quality through its GEF activity and ROCK

**Figure 5:**
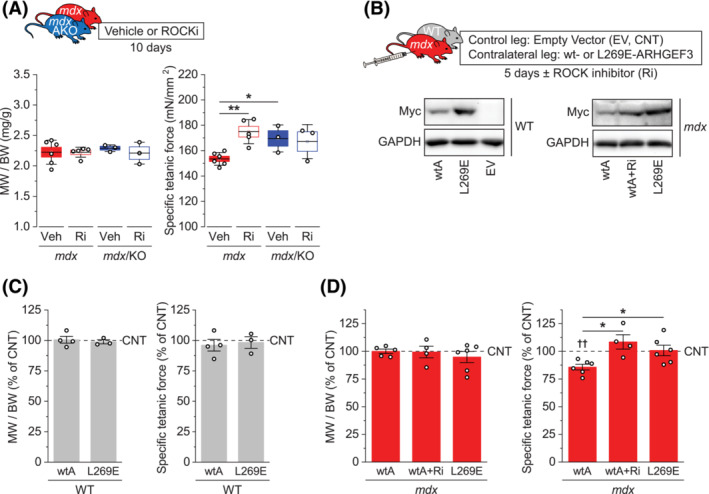
ARHGEF3 regulates *mdx* muscle quality through its GEF activity & ROCK. (A) Tibialis anterior (TA) muscles from 8‐week‐old *mdx* and *mdx*/AKO male mice treated with Y‐27632 (Ri) or vehicle (Veh) for 10 days were analysed for muscle weight (MW) to body weight (BW) ratio (left) and specific isometric tetanic force (right). (B) WT and *mdx* TA muscles of one mouse leg were transfected with Myc‐tagged WT ARHGEF3 (wtA) or GEF‐inactive ARHGEF3 (L269E), while contralateral TA muscles were transfected with empty vectors (EV) for internal control. The muscles were recovered for 5 days until 8‐week‐old in the presence or absence of Y‐27632 (Ri) treatment and analysed for protein expression of Myc‐tagged ARHGEF3 and GAPDH. (C, D) (C) WT and (D) *mdx* muscle samples from (B) were analysed for muscle weight (MW) to body weight (BW) ratio (left) and specific isometric tetanic force (right). Data are presented as mean ± SEM (box) and SD (whisker) (A) or ± SEM (C, D) with individual data points from each mouse. *P < 0.05, **P < 0.01 by two‐way ANOVA (A) or one‐way ANOVA (D). ††*P* < 0.01 versus contralateral controls by two‐tailed paired *t*‐test.

Our findings that ARHGEF3 depletion and ROCK inhibition similarly restored muscle quality and that activation of RhoA/ROCK signalling was dependent on ARHGEF3 in *mdx* muscles support the hypothesis that ROCK lies downstream of ARHGEF3 on the same pathologic pathway controlling *mdx* muscle quality. To further test this model, we treated *mdx* and *mdx*/AKO mice with Y‐27632. While ROCK inhibition and ARHGEF3 depletion separately improved specific muscle force in *mdx* mice without changing muscle mass, the combination of ROCK inhibition and ARHGEF3 KO did not exert any additive effect (Figure 5A). This observation suggests that ARHGEF3 and ROCK regulate muscle quality through the same or functionally redundant pathway.

Next, we transfected WT and *mdx* TA muscles of one mouse leg with plasmids expressing either WT Myc‐ARHGEF3 (wtA) or GEF‐inactive Myc‐ARHGEF3 (L269E)^15^ while transfecting contralateral TA muscles with empty vectors for internal control (Figure 5B). After 5 days, overexpression of ARHGEF3 did not alter muscle mass or specific muscle force in WT muscles regardless of its GEF activity (Figure 5C). On the other hand, in *mdx* muscles, overexpression of WT ARHGEF3 reduced specific muscle force with no effect on muscle mass, corroborating ARHGEF3's inhibitory function on *mdx* muscle quality (Figure 5D). Furthermore, this effect of ARHGEF3 was abrogated by the treatment of ROCK inhibitor or when GEF was inactive (Figure 5D). These results indicate that RhoA/ROCK signalling mediates ARHGEF3 regulation of muscle quality on the same pathway.

### ROCK signalling regulates *mdx* muscle quality via autophagy

**Figure 6:**
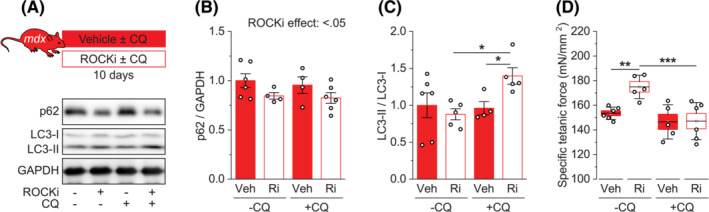
ROCK signalling regulates *mdx* muscle quality via autophagy. (A) Tibialis anterior (TA) muscles from *mdx* male mice treated with Y‐27632 (Ri) or vehicle (Veh) ± chloroquine (CQ) for 10 days were analysed for protein expression of p62, LC3, and GAPDH. (B, C) Quantification of (B) p62 to GAPDH ratio and (C) LC3‐II to LC3‐I ratio. (D) TA muscles treated as in (A) were analysed for specific isometric tetanic force. Data are presented as mean ± SEM (B, C) or ± SEM (box) and SD (whisker) (D) with individual data points from each mouse. *P < 0.05, **P < 0.01, ***P < 0.001 by two‐way ANOVA.

Having established the role of ARHGEF3/ROCK signalling in the control of *mdx* muscle quality, we wondered how this signalling regulates muscle quality in *mdx* mice. As mentioned earlier, changes in muscle quality in young *mdx* mice are attributable to changes in myofibre quality or force transmission. One key mechanism controlling myofibre force transmission involves autophagy, a homeostatic process essential for the removal of toxic cellular wastes. Previous studies have shown that autophagy is impaired in multiple types of muscular dystrophies, including DMD, and this contributes to muscular dysfunction.^26^, ^28^, ^29^ Studies have also reported that ROCK plays an important role in autophagy regulation in other conditions, although the role seems to vary depending on specific ROCK isoforms, cell types, and physiological conditions.^30^, ^31^, ^32^ Hence, we investigated the role that ROCK may play in autophagy in muscular dystrophy by treating *mdx* mice with Y‐27632 or a control vehicle with or without autophagy inhibitor chloroquine. We compared levels of p62/SQSTM1and LC3‐II/I ratio (LC3‐I lipidation) and their changes by the autophagy blocker, a method commonly used to access autophagy flux in skeletal muscles *in vivo*.^26^, ^33^ The level of p62 was significantly reduced by the ROCK inhibitor (Figures 6A,B). Because p62 protein (and LC3‐II) degrades through autophagy, this finding suggests that autophagic degradation of p62 or autophagy flux was enhanced by ROCK inhibition. Furthermore, chloroquine treatment significantly increased the level of LC3‐II/I ratio when ROCK was inhibited (Figures 6A,C), again suggesting enhanced autophagy flux with ROCK inhibition. Based on these results, we wanted to determine the role of autophagy in the ROCK‐dependent changes in *mdx* muscle quality. As shown in Figure 6D, inhibition of autophagy with chloroquine eliminated the Y‐27632‐induced increase in specific muscle force in *mdx* mice. Together, these findings indicate that ROCK negatively regulates autophagy and, thereby, *mdx* muscle quality.

### ARHGEF3 regulates *mdx* muscle quality via autophagy

**Figure 7:**
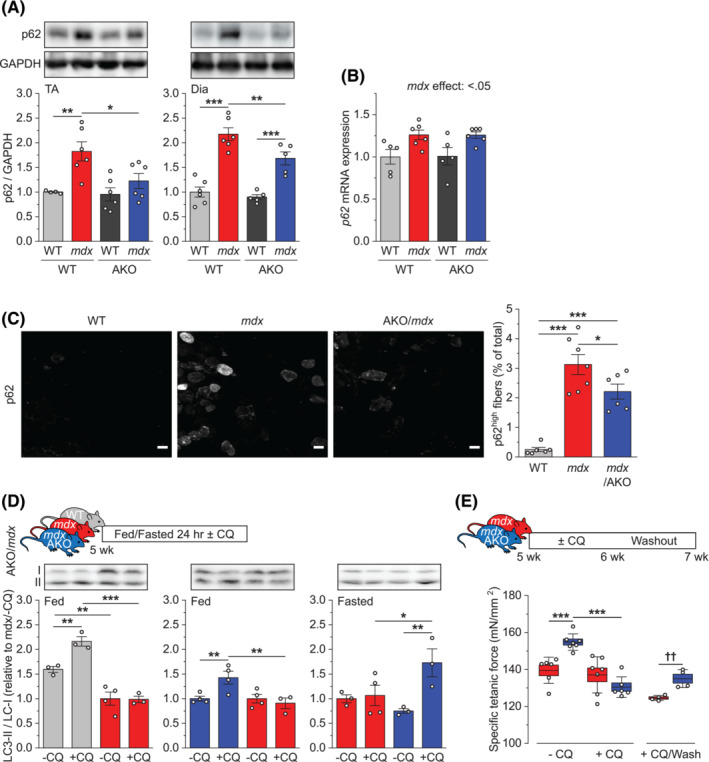
ARHGEF3 regulates *mdx* muscle quality via autophagy. (A‐B) tibialis anterior (TA) and/or diaphragm (Dia) muscles from 5‐week‐old WT, *mdx*, *Arhgef3* KO (AKO), and *mdx*/AKO male mice were analysed for (A) protein and (B) mRNA expression of p62. (C) TA muscle cross‐sections from 5‐week‐old WT, *mdx*, and *mdx*/AKO male mice were analysed for the proportion of the p62‐enriched myofibres. (D) Five‐week‐old WT, *mdx*, and *mdx*/AKO male mice were either fed or fasted for 24 h and treated with chloroquine or vehicle for 1 h. TA muscles were analysed for protein expression of LC3. (E) Five‐week‐old *mdx* and *mdx*/AKO male mice were treated with chloroquine or vehicle for 7 days, with some mice followed by 7 days of no drug treatment (washout). TA muscles were analysed for specific isometric tetanic force. Data are presented as mean ± SEM (A–D) or ± SEM (box) and SD (whisker) (E) with individual data points from each mouse. *P < 0.05, **P < 0.01, ***P < 0.001 by two‐way ANOVA. ††*P* < 0.01 by two‐tailed unpaired *t*‐test.

The role of ROCK in autophagy prompted us to perform the next set of experiments to test whether ARHGEF3 also regulates autophagy in *mdx* muscles. We found that depletion of ARHGEF3 reduced the level of p62 protein in *mdx* muscles without affecting p62 mRNA levels, which again suggests enhanced autophagic degradation of p62 (Figure 7A,B). Immunohistochemical analysis revealed that the ARHGEF3‐dependent changes in p62 occurred within myofibres as ARHGEF3 depletion also attenuated the population of the p62‐enriched myofibres in *mdx* muscles (Figure 7C). Interestingly, some of these myofibres contained p62‐positive aggregates, which is indicative of uncleared protein aggregates due to impaired autophagy.^34^ Furthermore, dystrophin deficiency in *mdx* muscles led to the prevention of the chloroquine‐induced increase in the ratio of LC3‐II/I even under the autophagy‐promoting fasting condition, but this ratio increased by chloroquine treatment in ARHGEF3‐depleted *mdx* muscles regardless of the feeding state (Figure 7D). The results of these experiments demonstrate that depletion of ARHGEF3 restores autophagy that is severely impaired in *mdx* muscles. Lastly, we examined a link between ARHGEF3‐regulated changes in autophagy and muscle quality. As shown in Figure 7E, ARHGEF3 depletion‐induced increase in specific muscle force was abolished by chloroquine treatment in *mdx* mice, and after 7 days of drug washout, the force‐promoting effect of ARHGEF3 depletion was restored. Taken together, our findings indicate that ARHGEF3 negatively regulates *mdx* muscle quality through autophagy.

## Discussion

Although DMD is caused initially by loss of dystrophin, the resulting pathological phenotypes, such as muscle weakness, are mediated by many molecular and cellular perturbations that are yet poorly characterized. In this study, we uncover a new pathologic pathway in which ARHGEF3 as a GEF activates RhoA/ROCK signalling, which in turn impairs muscle quality through impairing autophagy in *mdx* mice. These findings could increase our therapeutic opportunities and capability to treat DMD and potentially other muscular dystrophies.

Loss of muscle function or muscle weakness is the ultimate cause of low quality of life and premature death in DMD patients. Although previous studies suggested that inhibition of RhoA/ROCK signalling promotes myogenic differentiation and regeneration in a severe model of DMD (*mdx*/UKO),^9^, ^10^ the effect of inhibiting RhoA/ROCK signalling on muscle function was not well characterized. In the current study with an *mdx* model of DMD, we found evidence that inhibiting ROCK or its upstream activator ARHGEF3 can improve dystrophic muscle function by alleviating the loss of muscle quality, a characteristic feature of DMD observed in several model species.^35^, ^36^ We measured muscle quality (specific muscle force) in a single intact *mdx* muscle *in situ*, the gold standard for studying clinical consequences in DMD animal models.^35^ Given that gene editing‐based restoration of functional dystrophin also induced a comparable level of improvement of muscle quality with the same *in situ* measurement,^37^ we consider the beneficial effects observed in this study clinically relevant.

In this study, ROCK inhibition or ARHGEF3 depletion did not promote myogenic differentiation or muscle regeneration in the *mdx* condition. This was unexpected not just because previous studies showed beneficial effects of ROCK inhibition on myogenic differentiation and muscle regeneration in the *mdx*/UKO model^9^, ^10^ but because several studies also revealed a differentiation‐promoting effect of ROCK inhibition or ARHGEF3 knockdown in dystrophin‐expressing healthy myogenic cells.^15^, ^38^ In addition, depletion of ARHGEF3 has been shown to promote acute injury‐induced muscle regeneration, likely via ROCK, in WT mice at a wide range of ages.^12^ Thus, the pro‐myogenic or ‐regenerative effects of inhibiting ARHGEF3/ROCK signalling seemed to be conserved in different cellular contexts. The reason for this discrepancy is unclear, but several factors may have been involved, such as differences in the basal level of ARHGEF3/ROCK signalling, duration of drug treatment, culture conditions, and animal strain and age. Regardless, our results enabled us to identify the differentiation‐ or regeneration‐independent role of ARHGEF3/ROCK signalling in controlling dystrophic muscle quality. In the future, it will be interesting to test whether inhibition of ARHGEF3/ROCK signalling improves both muscle quality and regeneration in another model of DMD and thus exerts synergistic improvement of muscle function.

Another important finding in this study is that ARHGEF3/ROCK signalling was implicated in autophagy defects in *mdx* muscles. Autophagy impairment has been recognized as one of the leading secondary causes of muscle weakness in several types of muscular dystrophies.^26^, ^28^, ^29^ Indeed, our results showed that restoring autophagy via inhibition of ROCK or ARHGEF3 can alleviate the loss of *mdx* muscle quality. These findings are important when considering several benefits that can be obtained from autophagy‐based therapies. For instance, gene therapies aimed at restoring functional dystrophin possess several challenges, such as the need for the correction of more than 4000 different DMD mutations and the immune response against exogenous vectors and proteins.^39^, ^40^ Targeting autophagy, as a common modifier of DMD, can bypass these issues while effectively alleviating the disease severity. When combined with gene and/or other therapies, autophagy correction can also improve the overall efficacy of DMD treatment. Hence, our findings that shed light on how we may correct autophagy in DMD have significant translational implications for DMD treatment. Furthermore, the mechanisms identified in our study can be explored as potential targets for several other autophagy‐dependent muscular dystrophies.

While preclinical animal studies allow various ways of accessing contractile muscle function to evaluate therapeutic candidates, it is not possible to perform such functional evaluations in conventional *in vitro* cell culture systems. This has been a major barrier to conducting translationally relevant DMD studies *in vitro*, which otherwise would provide convenient, low‐cost, and animal‐free drug screening and validation along with mechanistic insights. In this study, by engineering 3D *in vitro* skeletal muscle from *mdx* cells, which accurately recapitulated the contraction defect in *mdx* muscles *in vivo*,^7^ we were able to test a direct action of ROCK inhibitor on *mdx* muscle cells and reveal its potential efficacy in improving *mdx* muscle contractility. This *in vitro* system can be easily extended to any type of skeletal muscle cells, including a patient's own cells, to develop a personalized gene and cell therapy for DMD.^39^ This potential application is especially attractive when considering the well‐known heterogenic treatment effects in patients carrying even the same mutation.^39^ The 3D *in vitro* system will strengthen our ability to develop therapeutic strategies for treating contractile dysfunction and muscle weakness in DMD.

## Conflict of interest statement

All authors declare that they have no conflict of interest.

## Supporting information


**Figure S1.** CAD design and the dimension of the 3D‐printed cantilever structure from (A) front, (B) side, and (C) top views. Unit: mm.
**Figure S2.** The relative abundance of *Arhgef3*, *Ect2*, *Farp1*, and *Vav2* transcripts was calculated using 2^—∆Ct^ in the tibialis anterior muscles of 5‐week‐old WT male mice. Data are presented as mean ± SEM.
**Figure S3.** Body weights of *mdx* and *mdx*/ARHGEF3 KO male mice. Data are presented as mean ± SEM with individual data points from each mouse.
**Figure** **S4.** Depletion of ARHGEF3 improves muscle function in female *mdx* mice. Tibialis anterior muscles from *mdx* and *mdx*/AKO female mice were analysed for muscle weight (MW) to body weight (BW) ratio (left) and specific isometric tetanic force (right). Data are presented as mean ± SEM (box) and SD (whisker) with individual data points from each mouse. **P < 0.01 by 2‐tailed unpaired t‐test.
**Figure S5.** Depletion of ARHGEF3 does not affect injury susceptibility in *mdx* muscles. Tibialis anterior muscles from WT, Arhgef3 KO (AKO), *mdx,* and *mdx*/AKO male and female mice were analysed for isometric tetanic force in‐between every two eccentric contractions (*n* = 5–7 mice per group). Data are presented as mean ± SEM.
**Figure S6.** Depletion of ARHGEF3 does not influence the expression of inflammatory markers in *mdx* muscles. Tibialis anterior muscles from 5‐week‐old WT, *mdx*, Arhgef3 KO (AKO), and *mdx*/AKO male mice were analysed for mRNA expression of several inflammatory markers (*n* = 5–7 mice per group). Data are presented as mean ± SEM. No significant effect of AKO was detected by 2‐way ANOVA.
**Figure S7.** An example of Open‐CSAM analysis of muscle fibre cross‐sectional area in the entire *mdx* TA muscle cross‐section. Any muscle fibres that were not or falsely measured by Open‐CSAM were manually corrected by investigators blinded to the sample identification.
**Table S1.** A list of primers used for qPCR.

**Movie S1.** Supporting Information.

**Movie S2.** Supporting Information.
