# Supplementary material for: RhoA/ROCK signalling activated by ARHGEF3 promotes muscle weakness via autophagy in dystrophic mdx mice
Source: J Cachexia Sarcopenia Muscle. 2023 Jun 13;14(4):1880–93. doi: 10.1002/jcsm.13278 (PMC10401546; doi:10.1002/jcsm.13278)
Supplement: Supplementary file 1 — Figure S1. CAD design and the dimension of the 3D‐printed cantilever structure from (A) front, (B) side, and (C) top views. Unit: mm. Figure S2. The relative abundance of Arhgef3, Ect2, Farp1, and Vav2 transcripts was calculated using 2—∆Ct in the tibialis anterior muscles of 5‐week‐old WT male mice. Data are presented as mean ± SEM. Figure S3. Body weights of mdx and mdx/ARHGEF3 KO male mice. Data are presented as mean ± SEM with individual data points from each mouse. Figure S4. Depletion of ARHGEF3 improves muscle function in female mdx mice. Tibialis anterior muscles from mdx and mdx/AKO female mice were analysed for muscle weight (MW) to body weight (BW) ratio (left) and specific isometric tetanic force (right). Data are presented as mean ± SEM (box) and SD (whisker) with individual data points from each mouse. **P < 0.01 by 2‐tailed unpaired t‐test. Figure S5. Depletion of ARHGEF3 does not affect injury susceptibility in mdx muscles. Tibialis anterior muscles from WT, Arhgef3 KO (AKO), mdx, and mdx/AKO male and female mice were analysed for isometric tetanic force in‐between every two eccentric contractions (n = 5–7 mice per group). Data are presented as mean ± SEM. Figure S6. Depletion of ARHGEF3 does not influence the expression of inflammatory markers in mdx muscles. Tibialis anterior muscles from 5‐week‐old WT, mdx, Arhgef3 KO (AKO), and mdx/AKO male mice were analysed for mRNA expression of several inflammatory markers (n = 5–7 mice per group). Data are presented as mean ± SEM. No significant effect of AKO was detected by 2‐way ANOVA. Figure S7. An example of Open‐CSAM analysis of muscle fibre cross‐sectional area in the entire mdx TA muscle cross‐section. Any muscle fibres that were not or falsely measured by Open‐CSAM were manually corrected by investigators blinded to the sample identification. Table S1. A list of primers used for qPCR. [file JCSM-14-1880-s003.zip › JCSM13278_Supporting information_rev.doc]

***Journal of Cachexia, Sarcopenia and Muscle***

**RhoA/ROCK signaling activated by ARHGEF3 contributes to autophagy-dependent muscle weakness in** **Duchenne muscular dystrophy**

Jae-Sung You^1,2,3,*^, Yongdeok Kim^3,4^, Soohyun Lee^1^, Rashid Bashir^2,3,4,5,6^, and Jie Chen^1,*^

^1^Department of Cell and Developmental Biology, University of Illinois at Urbana-Champaign, Urbana, IL 61801.

^2^Department of Bioengineering, University of Illinois at Urbana–Champaign, Urbana, IL 61801.

^3^Nick J. Holonyak Micro and Nanotechnology Laboratory, University of Illinois at Urbana–Champaign, Urbana, IL 61801.

^4^Department of Materials Science and Engineering, University of Illinois at Urbana–Champaign, Urbana, IL 61801.

^5^Department of Mechanical Science and Engineering, University of Illinois at Urbana–Champaign, Urbana, IL 61801.

^6^Department of Biomedical and Translational Sciences, Carle Illinois College of Medicine, Urbana, IL 61801.

* Correspondence to:

Jae-Sung You, Email: [bockgiri@gmail.com](mailto:youj@illinois.edu).

Jie Chen, Email: [jiechen@illinois.edu](mailto:jiechen@illinois.edu).

Figure S1. CAD design and the dimension of the 3D-printed cantilever structure from (A) front, (B) side, and (C) top views. Unit: mm

**Figure S2.** The relative abundance of *Arhgef3*, *Ect2*, *Farp1*, and *Vav2* transcripts was calculated using 2^—∆Ct^ in the tibialis anterior muscles of 5-week-old WT male mice. Data are presented as mean ± SEM.

Figure S3. Body weights of *mdx* and *mdx*/ARHGEF3 KO male mice. Data are presented as mean ± SEM with individual data points from each mouse.

Figure S4. Depletion of ARHGEF3 improves muscle function in female mdx mice. Tibialis anterior muscles from *mdx* and *mdx*/AKO female mice were analyzed for muscle weight (MW) to body weight (BW) ratio (left) and specific isometric tetanic force (right). Data are presented as mean ± SEM (box) and SD (whisker) with individual data points from each mouse. **P < 0.01 by 2-tailed unpaired t-test.

Figure S5. Depletion of ARHGEF3 does not affect injury susceptibility in *mdx* muscles. Tibialis anterior muscles from WT, Arhgef3 KO (AKO), *mdx,* and *mdx*/AKO male and female mice were analyzed for isometric tetanic force in-between every two eccentric contractions (n = 5-7 mice per group). Data are presented as mean ± SEM.

Figure S6. Depletion of ARHGEF3 does not influence the expression of inflammatory markers in *mdx* muscles. Tibialis anterior muscles from 5-week-old WT, *mdx*, Arhgef3 KO (AKO), and *mdx*/AKO male mice were analyzed for mRNA expression of several inflammatory markers (n = 5-7 mice per group). Data are presented as mean ± SEM. No significant effect of AKO was detected by 2-way ANOVA.

Figure S7. An example of Open-CSAM analysis of muscle fiber cross-sectional area in the entire *mdx* TA muscle cross-section. Any muscle fibers that were not or falsely measured by Open-CSAM were manually corrected by investigators blinded to the sample identification.

**Table S1.** A list of primers used for qPCR.

| **Gene** | **Forward (5’-3’)** | **Reverse (5-’3’)** |
| --- | --- | --- |
| ***Arhgef3*** | CTTCAGCAACCACGAGAGAGT | GAAGGTGTCGTTGGCTTGTA |
| ***Arhgef5*** | CCCAGTGTCTTGGCATTCTT | AGGGAAAGCAGAGGAAGCTC |
| ***Arhgef10*** | GCAGAGAAGCTAAACGAGAGAA | CTGAGGAGCTTGTTCAGGTATC |
| ***Arhgef12*** | CCCGACAAGCTCTCAGATAAA | AGCCAGGATGTGGTTGATAC |
| ***Arhgef18*** | GAGTTCCAGGTCTACCACATTT | TTGGTCCTGCCTGTTGTTAG |
| ***Mcf2*** | CCAACAGGCTGGTTGTAAGA | TATCATTCTGGACCGCATCAC |
| ***Arhgef25*** | GATGGCTCTGGAAAGGAGTATG | TGGTAGCCATGTAACCCTCTA |
| ***Ect2*** | GAAAGCGGCACAAGGTTATTG | CTGAGAAAGAGGCATGAGATGAA |
| ***Arhgef40*** | TGAAGTCCCTCCTCCATTCT | GCCCGAGGACATCATAGATAAG |
| ***Farp1*** | CCATGCCTTCTTCAGACTCTTC | CTTCTGAGTCCGACCACTAAAC |
| ***Plekhg6*** | CCCGACAAGCTCTCAGATAAA | AGCCAGGATGTGGTTGATAC |
| ***Bcr*** | GGGATAGGAGCAAGATGGAAAG | TAACTAGGGTGGCCAGTAAGA |
| ***Vav2*** | GAGCTTAAAGTCCGGTCCATAG | TAGCTGTAGCCCTTCCTCTT |
| ***Sqstm1/p62*** | CCCAGTGTCTTGGCATTCTT | AGGGAAAGCAGAGGAAGCTC |
| ***Adgre1*** | ACAGTCATCTCCCTGGTATGT | TGCAGGTGCATGTAGGTATTG |
| ***Cd74*** | CCTCAAGGAAGAAGAACCCAAG | GCTGAGCAAGGAACCTGAAA |
| ***Nos2*** | CTTGGAGCGAGTTGTGGATT | CTCTTGTCTTTGACCCAGTAGC |
| ***Il-6*** | CTTCCATCCAGTTGCCTTCTT | ATCCTCTGTGAAGTCTCCTCTC |
| ***Ifng*** | TCCTTTGGACCCTCTGACTT | AAGCCAAGATGCAGTGTGTAG |
| ***Tnf*** | GGGAGAACAGAAACTCCAGAAC | CAGTGAGTGAAAGGGACAGAAC |
| ***Il-10*** | CTATGCTGCCTGCTCTTACTG | GGGAAGTGGGTGCAGTTATT |
| ***Il-4*** | GACGGCACAGAGCTATTGAT | GGATATGGCTCCTGGTACATTC |
| ***Gapdh*** | GCAGCCTCGTCCCGTAGAC | ATGGCAACAATCTCCACTTTGC |

**Supplementary Materials and Methods**

**Antibodies**

The antibodies used in this study are anti-ARHGEF3 (Khanna et al., 2013); anti-GAPDH (Cat# 2118, RRID: AB_561053), anti-RhoA (Cat# 2117, RRID: AB_10693922), anti-pSer473-Akt (Cat# 9271, RRID: AB_329825), anti-Akt (Cat# 9272, RRID: AB_329827), anti-p62 (Cat# 23214, RRID: AB_2798858), and anti-LC3A/B (Cat# 4108, RRID: AB_2137703) from Cell Signaling Technology; anti- myosin heavy chain (MHC), sarcomere (Cat# MF-20, RRID:AB_2147781) from Developmental Studies Hybridoma Bank; anti-laminin (Cat# L9393, RRID:AB_ 477163) from Sigma-Aldrich; anti-c-myc (Cat# MMS-150R-1000, Clone 9E10, RRID:AB_291325) from Covance; peroxidase-conjugated anti-rabbit (Cat# 111-036-003, RRID:AB_2337942) and anti-mouse (Cat# 115-036-003, RRID:AB_2338518) IgG from Jackson Immuno Research Laboratories; Alexa Fluor 488–conjugated anti-mouse IgG (Cat# A-11001, RRID:AB_2534069) and Alexa Fluor 594–conjugated anti-rabbit IgG (Cat# A-11012, RRID:AB_2534079) from Thermo Fisher Scientific.

**Immunohistochemistry**

Ten-µm-thick cross-sections were obtained from the mid-belly of the TA muscle frozen in optimal cutting temperature compound using a cryostat and fixed in acetone for 10 min at -30°C. The sections were rehydrated with PBS for 15 min, incubated in solution A (0.5% BSA and 0.5% Triton X-100 in PBS) containing 5% normal goat serum (Jackson ImmunoResearch) for 1 hour, and probed with anti-laminin or -p62 antibody in solution A for 1 hour. After washing with PBS, the sections were incubated with the appropriate fluorophore-conjugated secondary antibody in solution A for 1 hour, followed by incubation with DAPI (5 µg/ml) for 5 min when needed. After a final wash with PBS, fluorescent signals from the entire cross-section were magnified by a Fluotar 4X objective on a Leica DMI 4000B microscope (Leica) and captured with a Retiga EXi camera (QImaging) and Image Pro Express software (Media Cybernetics). All images were analyzed by investigators blinded to the sample identification using ImageJ (https://imagej.nih.gov/ij/). The cross-sectional area and the total number of myofibers were measured semi-automatically by Open-CSAM software^1^.

**Immunofluorescence**

MPCs differentiated in 12-well plates were fixed with 3.7% formaldehyde for 15 min, incubated in solution B (0.1% Triton X-100 and 3% BSA in PBS) for 10 min, and probed with anti-MHC antibody in solution B for 1 hour. After washing with PBS, the cells were incubated with the appropriate fluorophore-conjugated secondary antibody and DAPI in solution B for 30 min. Fluorescence images were captured and analyzed as described above using a 20X objective and ImageJ. MHC^+^ area was measured automatically using the Huang threshold. The fusion index was calculated as the percentage of nuclei in MHC^+^ cells with more than one nucleus. Each data point was generated by averaging nine representative microscopic fields.

**Western Blotting**

Frozen muscles were homogenized with a Polytron in ice-cold buffer [50 mM Tris (pH 7.4), 0.5% sodium deoxycholate, 0.1% SDS, 1% Triton X-100, 500 mM NaCl, 10 mM MgCl_2_, 0.1 mM Na_3_VO_4_, 25 mM NaF, 25 mM β-glycerolphosphate, and 1× protease inhibitor cocktail (P8340, Sigma-Aldrich)], and the homogenates were centrifugated at 16,200 g for 10 min (4°C). After adjusting the protein concentration in the supernatants equal, the samples were combined with 2X Laemmli buffer. For *in vitro* samples, cells were directly lysed with 1X Laemmli buffer and sonication. Western blotting was performed following the standard procedure as described previously^2^, using an iBright CL1000 Imaging System (Thermo Fisher Scientific) for a wide linear range of detection and image J for quantification.

**RhoA activity assay**

To pull down the GTP-bound active form of RhoA, an equal amount of protein from each muscle sample prepared as above was incubated with 20 µl Rhotekin-RBD beads (Cytoskeleton) for 2 hours at 4°C. The beads were washed 4 times in ice-cold buffer [50 mM Tris (pH 7.4), 1% Triton X-100, 150 mM NaCl, 10 mM MgCl2, and 1× protease inhibitor cocktail], boiled in 1X Laemmli buffer, and subjected to Western blotting with anti-RhoA primary antibody. When determining RhoA-specific GEF activity, the amount of GTP-bound RhoA was normalized to the amount of total RhoA.

**Quantitative PCR**

Frozen muscles were homogenized with a ribonuclease-free pestle in ice-cold TRIzol (Invitrogen). Total RNA was extracted and reverse transcribed using the GeneJET RNA Purification Kit (Thermo Fisher Scientific) and the qScript cDNA Synthesis Kit (Quanta Bioscience), respectively. Complementary DNA was subjected to qPCR on a StepOnePlus Real-Time PCR System (Applied Biosystems) using SYBR Green. The primer sequences used are listed in Table S1.

**Supplementary References**

1.	Desgeorges T, Liot S, Lyon S, Bouvière J, Kemmel A, Trignol A *et al.* Open-CSAM, a new tool for semi-automated analysis of myofiber cross-sectional area in regenerating adult skeletal muscle. *Skelet Muscle* 2019;**9**:2.

2.	You J-S, Chen J. Aging Does Not Exacerbate Muscle Loss During Denervation and Lends Unique Muscle-Specific Atrophy Resistance With Akt Activation. *Front Physiol* 2021;**12**.
